# Estimation of the HIV-1 backward mutation rate from transmitted drug-resistant strains

**DOI:** 10.1016/j.tpb.2016.08.001

**Published:** 2016-12

**Authors:** J.M. Kitayimbwa, J.Y.T. Mugisha, R.A. Saenz

**Affiliations:** a Department of Mathematics, Makerere University, P.O. Box 7062, Kampala, Uganda; b Department of Computing and Technology, Uganda Christian University, P.O. Box 4, Mukono, Uganda; c Facultad de Ciencias, Universidad de Colima, Bernal Díaz del Castillo 340, Colima, COL, C.P. 28045, Mexico

**Keywords:** Backward mutation rate, Within-host model, HIV-1, Virus mutations, Kaplan–Meier estimates

## Abstract

One of the serious threats facing the administration of antiretroviral therapy to human immunodeficiency virus (HIV-1) infected patients is the reported increasing prevalence of transmitted drug resistance. However, given that HIV-1 drug-resistant strains are often less fit than the wild-type strains, it is expected that drug-resistant strains that are present during the primary phase of the HIV-1 infection are replaced by the fitter wild-type strains. This replacement of HIV-1 resistant mutations involves the emergence of wild-type strains by a process of backward mutation. How quickly the replacement happens is dependent on the class of HIV-1 mutation group.

We estimate the backward mutation rates and relative fitness of various mutational groups known to confer HIV-1 drug resistance. We do this by fitting a stochastic model to data for individuals who were originally infected by an HIV-1 strain carrying any one of the known drug resistance-conferring mutations and observed over a period of time to see whether the resistant strain is replaced. To do this, we seek a distribution, generated from simulations of the stochastic model, that best describes the observed (clinical data) replacement times of a given mutation. We found that Lamivudine/Emtricitabine-associated mutations have a distinctly higher, backward mutation rate and low relative fitness compared to the other classes (as has been reported before) while protease inhibitors-associated mutations have a slower backward mutation rate and high relative fitness. For the other mutation classes, we found more uncertainty in their estimates.

## 1 Introduction

Administration of antiretroviral therapy (ART) to HIV-1 patients has been greatly improved through the use of at least three drugs in combination. However, treatment failure is reported in case of poor adherence or drug toxicities with some patients developing detectable viral loads during the course of ART. This is associated with the emergence of drug resistance to one or more drugs in the drug cocktail. Therefore, life-long ART together with emergence of drug resistance has resulted into an ever increasing pool of individuals who can transmit HIV-1 drug resistant strains (Cane, 2005). Mutations in the HIV-1 genome that confer resistance during ART have been detected in plasma and cellular reservoirs of ART-naïve HIV-1 infected patients worldwide (Geretti, 2007). Several of these are recognized markers of primary or transmitted drug resistance (TDR).

The time of testing in relation to the time of infection and the sensitivity of the resistance testing methods are crucial for proper detection of TDR. TDR has been mainly reported in resource-rich settings where the coverage of ART is extensive. It is estimated that 10%–20% of new diagnoses in Europe and the USA with HIV-1 are resistant to at least one drug. However, the reported prevalence of TDR remains low in resource-poor settings, where the ART coverage is still sub-optimal (Cane, 2005, Hamers et al., 2011). One cause for concern in resource-limited settings is that administration of ART and switching of regimens are still based on clinical criteria since availability of assays for monitoring patients on ART is limited and access to resistance testing is still not possible. Thus, a person continues treatment on a failing regimen for a longer period of time. This means that resistant virus can continue replication under drug pressure, increasing the risk of onward transmission of drug-resistant HIV-1 (Geretti, 2007). Reports indicate that 75%–80% of patients undergoing ART with detectable plasma HIV-1 RNA levels carry viruses with reduced susceptibility to one or more drugs (Richman et al., 2004). Such individuals are an obvious risk group of transmitting drug resistant HIV-1 (Pingen et al., 2011).

Resistant virions that arise during ART are quickly replaced by wild-type strains on cessation or interruption of treatment (Kitayimbwa et al., 2013, Vaidya et al., 2010, McLean and Nowak, 1992). However, unlike resistance-conferring mutations that are acquired during ART, transmitted drug resistant HIV-1 can persist long term in the absence of drug pressure (Little et al., 2008, Pingen et al., 2011). One possible explanation of this occurrence is that soon after infection, a homogenous viral population is established allowing for no effective competition with wild-type virus. A mutation of resistant virus to wild-type (commonly referred to as a backward mutation) may occur over time, although in some cases the resistant virions become genetically fixed by compensatory mutations and unable to revert to wild-type without a loss of fitness (Little et al., 2008, Geretti, 2007, De Ronde et al., 2001).

The mutational process of HIV-1 within a host is in essence the result of a stochastic process in which a single mutation appears more frequently than double or multiple mutations (Keulen et al., 1996). Emergence of a fitter viral strain with a higher replicative capacity leads to overgrowth of the parent viral strain (McLean and Nowak, 1992). Thus, this process of selection results in the appearance of mutants with the highest fitness in a given environment. A stochastic model is then suitable for such a process since, even though all possibilities of what happens when a person is infected with a resistant strain cannot be generated, individual simulations form part of an ensemble of possibilities, i.e., a collection of micro states of the system. We use this ensemble to visualize the probability distribution over the set of micro states.

In this study, we estimate the backward mutation rates and relative fitness of various mutational groups known to confer HIV-1 drug resistance. We do this by fitting a stochastic model to data for individuals who were originally infected by an HIV-1 strain carrying any one of the known drug resistance-conferring mutations and observed over a period of time to see whether the resistant strain is replaced. To do this, we seek a distribution (generated from simulations of the stochastic model) that best describes the replacement times of a given mutation, from clinical data, using the Kaplan–Meier estimation of the survival function.

## 2 Methods

### 2.1 Datasets

Clinical data for individuals with acute and/or early HIV-1 infection enrolled in two prospective studies, taken from supplementary information in  Jain et al. (2011), were used. The two prospective cohort studies were the Options Project (San Francisco General Hospital, University of California, San Francisco [UCSF]) and an acute and/or early HIV-1 infection cohort in Sao Paulo, Brazil.

The Options Project was a prospective cohort study in which individuals were enrolled within 12 months after HIV-1 antibody sero-conversion (restricted to 6 months after sero-conversion). Participants were enrolled after screening for acute and/or early HIV-1 infection. In the case of the Sao Paulo cohort, patients who had experienced seroconversion within the previous 6 months and had evidence of acute and/or early HIV-1 infection were recruited. For both studies, participants were ART-naïve patients who presented TDR on initial genotyping, with 6 or more months of follow-up without ART and with one or more follow-up genotype. Only mutations that are known to be selected by ART were considered with common polymorphic mutations excluded (Jain et al., 2011).

In both sets of data, follow-up genotypes were obtained, every 3–4 months, to estimate the time at which baseline TDR mutations became undetectable by population sequencing. For individuals that started ART, the last available specimen before ART initiation was genotyped while for ART-naïve patients, the last available specimen was genotyped. For individuals with baseline TDR mutations still present at the last time point, it was noted that replacement of resistant mutations had not occurred. However, if no baseline TDR mutations were detected at the final time point, specimens closest to the midpoint of the prior 2 specimens was genotyped.

The drug resistance-conferring mutations studied are grouped into six different categories:

(i) lamivudine/emtricitabine-associated mutations M184V/I
(ii) thymidine analog-associated (TAM) mutations M41L, D67N, K70R, L210W, T215Y/F, and K219Q/E
(iii) T215 partial revertant mutations T215C, T215D, T215E, T215I, T215S and T215V
(iv) other nucleoside reverse-transcriptase inhibitor (NRTI) mutations
(v) nonnucleoside reverse-transcriptase inhibitor (NNRTI) mutations
(vi) protease inhibitor (PI) mutations.

Details of these categories can be found in  Jain et al. (2011).

### 2.2 Modeling framework

#### 2.2.1 The mean-field dynamics

We model HIV-1 dynamics within a host by considering explicitly the concentrations of the uninfected target cells T, cells infected with the sensitive strain $I_{s}$, cells infected with the resistant strain $I_{r}$, sensitive virus $V_{s}$ and resistant virus $V_{r}$. System  (1) describes the interactions between these various cell and viral populations. System  (1) is similar to the one described in  Kitayimbwa et al. (2013) but with the forward mutations (i.e., mutations from the sensitive to the resistant strains) considered to be negligible since there is no drug pressure.

(1) $$\frac{dT}{dt}=\lambda -\gamma T-\beta TV_{s}-k_{1}\beta TV_{r}$$

$$\frac{dI_{s}}{dt}=\beta TV_{s}+zk_{1}\beta TV_{r}-\delta I_{s}$$

$$\frac{dI_{r}}{dt}=\left(1-z\right)k_{1}\beta TV_{r}-\delta I_{r}$$

$$\frac{dV_{s}}{dt}=aI_{s}-cV_{s}$$

$$\frac{dV_{r}}{dt}=k_{2}aI_{r}-cV_{r}\text{.}$$

**Fig. 1:**
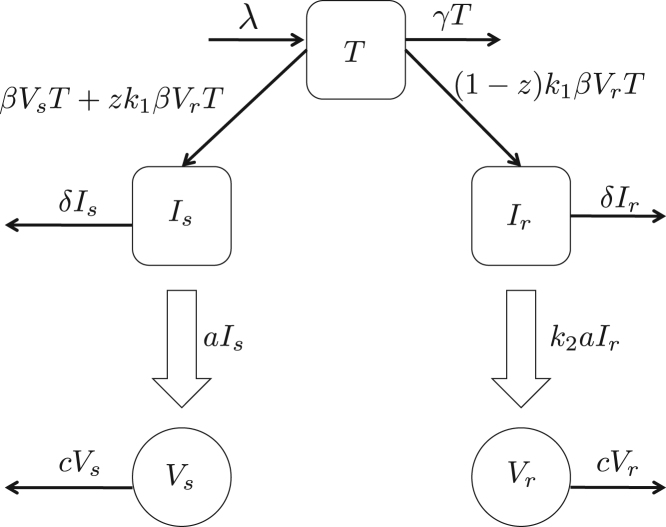
Schematic diagram showing infection dynamics of System (1). CD4^+^ T-cells are classified into uninfected T and infected with ART-sensitive or ART-resistant virus, $I_{s}$ and $I_{r}$, respectively. Virus strains are either sensitive, $V_{s}$, or resistant, $V_{r}$, to ART. See text for details.

In System  (1), the target cells T are constantly recruited from the thymus at a rate λ and die at a rate γ. Since the drug-resistant strain is generally less fit than the sensitive strain, resistant virus infects target cells at a rate $k_{1}\beta$ where β is the rate of infection of the target cells by the sensitive strain and $k_{1}\in \left(0,1\right)$ is the relative fitness of the resistant strain on infectivity. Due to the HIV-1 replication process being error-prone, resistant virus mutates to sensitive virus at a rate z (backward mutation acts as the source for any arising sensitive strain). Cells infected by either resistant or sensitive virus die at an infection-induced death rate δ. Cells infected with sensitive virus release new virions at a rate a. The cells infected with resistant virus release new virions at rate $k_{2}a$ where $k_{2}\in \left(0,1\right)$ is the relative fitness of the resistant virus in terms of viral productivity. Resistant and sensitive virus is cleared from the system at a rate c. Fig. 1 shows the flow diagram of the compartmental deterministic model as described above. In the limit of large population numbers present in all compartments, the HIV-1 within-host dynamics for a transmitted drug-resistant strain can be described by the mean field equations in System  (1). Such a system neglects all the stochastic aspects present within a host and only focuses on the evolution of the mean population numbers.

#### 2.2.2 Analysis of the mean-field dynamics

The System  (1) has two feasible equilibrium points:

(i) The infection-free equilibrium
  $$E_{0}=\left(\frac{\lambda }{\gamma },0,0,0,0\right)\text{.}$$
(ii) The sensitive strain dominated equilibrium $E_{1}=\left(T^{*},I_{s}^{*},0,V_{s}^{*},0\right)$ where
  $$T^{*}=\frac{\lambda }{\gamma R_{0}}$$
  $$I_{s}^{*}=\frac{\gamma c}{\beta a}\left(R_{0}-1\right)$$
  $$V_{s}^{*}=\frac{\gamma }{\beta }\left(R_{0}-1\right)$$
  where
  $$R_{0}=\frac{\lambda \beta a}{\gamma \delta c}\text{.}$$

Therefore, whenever $R_{0}>1$, there exists a unique endemic equilibrium $E_{1}$ with no resistant virus present. From the definition of $E_{1}$, it is observed that drug resistant strains will always be replaced as long as the backward mutation is positive (i.e.,  z>0). The fact that a resistant strain is always replaced is due to the assumption that mutation only occurs in one direction, from the resistant to the sensitive strain, and the fitness cost of drug resistance. If forward mutations (i.e., mutations from sensitive to resistant strains) are also included, there will be coexistence of strains with the dominant strain determined by the competition between the two strains (Kitayimbwa et al., 2013, McLean and Nowak, 1992, Rong et al., 2007, Vaidya et al., 2010).

The following stability results can be easily proved by linearizing System  (1).

Proposition 2.1 *The infection free steady state* $E_{0}$ *is locally asymptotically stable if* $R_{0}<1$ *and it is unstable if* $R_{0}>1$*.*

Proposition 2.2 *The endemic equilibrium* $E_{1}$ *exists if and only if* $R_{0}>1$*.* $E_{1}$ *is locally asymptotically stable whenever it exists.*

Therefore, for all parameter values, whenever $R_{0}>1$, the endemic equilibrium $E_{1}$ is locally asymptotically stable. We would therefore expect that given enough time, all TDR viral strains would be replaced by the sensitive strain.

### 2.3 Stochastic formulation for the population dynamics

The ODEs described by System  (1) can be numerically integrated to obtain the mean-field population evolution. So long as the population size of each compartment is large enough, stochastic changes are negligible. For consideration of transmitted drug resistance, we assume that an individual is initially infected with a resistant strain. Subsequent replacement of the resistant strain by a sensitive strain requires de-novo mutations within the host. Therefore, the replacement of the resistant strain is expected to be greatly affected by stochastic changes. For this reason, the analysis of the replacement times for the resistant strain by the sensitive strain for HIV-1 patients initially infected by a resistant strain requires a stochastic analysis. Such an analysis provides not only the mean-field evolution of the population numbers but also the evolution of its variability. In a deterministic setting, one would expect that with time, all the drug resistant strains would be replaced by the sensitive strain because of differences in fitness. However, in a stochastic setting, variability plays a key role at very low numbers. In this case, not all resistant strains are observed to be replaced in our period of observation.

#### 2.3.1 Stochastic model formulation

To study the replacement times of HIV-1 drug resistance-conferring mutations, we develop a stochastic model. The model used is the stochastic version of the deterministic model given by System (1). The stochastic model tracks discrete changes over time in population sizes for the target cells (T), cells infected with sensitive virus $\left(I_{s}\right)$, cells infected with resistant virus $\left(I_{r}\right)$, sensitive virus $\left(V_{s}\right)$, and resistant virus $\left(V_{r}\right)$. We express changes in $T,\;I_{s},\;I_{r},\;V_{s}$ and $V_{r}$ in an arbitrarily small interval (t,t+Δt) as $\Delta T,\Delta I_{s},\Delta I_{r},\Delta V_{s}$, and $\Delta V_{r}$, respectively. Here, a stochastic process is defined by the probabilities at which different events occur in the time period Δt. The possible events for the various populations modeled are as follows: recruitment of CD4^+^ cells and production of new HIV-1, death (CD4^+^ and HIV-1), and infection of target cells by either virus (including mutations). The probabilities that any of the above events occur in the time interval (t,t+Δt) are given by their corresponding rates in System  (1). Table 1 shows the rates for the various events in the model with total rate $\Phi =\lambda +\gamma T+\beta TV_{s}+k_{1}\beta TV_{r}+\delta I_{s}+\delta I_{r}+aI_{s}+k_{2}aI_{r}+cV_{s}+cV_{r}$ as the scaling factor guaranteeing that all the probabilities are between 0 and 1.

#### 2.3.2 Parameter values

For all simulations, we use parameter values as outlined in Table 2. We make the assumption that the resistant virus is just as infectious as the sensitive strain $\left(k_{1}=1\right)$ and only account for its fitness disadvantage at the viral production stage of its life cycle (varying $k_{2}$). If on the other hand, we fix $k_{2}=1$ and vary $k_{1}$, very similar results would be obtained. This is not surprising since the combined effect on fitness at both viral production and infection stages of the HIV-1 life cycle is multiplicative $\left(k=k_{1}k_{2}\right)$. Therefore, for this work, we only estimate $k_{2}$.

#### 2.3.3 Simulations

In order to speed up the simulations, the stochastic model is implemented using the τ-leap method (Gillespie, 2001). The virus–cell dynamics are simulated effectively in a volume of 1 ml, as in previous studies (Cadosh et al., 2012, Pearson et al., 2011, Ribeiro and Bonhoeffer, 2000). In a volume of 1 ml of blood, the target population size was taken as 10^4^ cells. Such a large census population size does not imply the absence of stochastic effects during the evolution of HIV-1 within an infected host. For the study of replacement times for resistance-conferring mutations, the effective population size, $N_{e}$, is indeed considerably smaller than the census population size (Kouyos et al., 2006).

Every run starts with a population of target cells at the carrying capacity $\left(T_{0}=\lambda /\gamma \right)$, an inoculum of 500 resistant virus, 10 T-cells infected with resistant virus, and no sensitive virus at all at the beginning assuming that initially, all infections are by a resistant strain, i.e., transmitted drug resistance. A simulation is allowed to run for a maximum simulation time of 3000 days, which covers all available clinical data. If the viral load for the resistant strain falls below the detection limit of 50, the simulation is stopped and the time at which this happens is recorded. This time is then considered as the replacement time for the resistance-conferring mutation. For the simulations that run for the entire 3000 days, we know that the drug-resistant strain survives up to the end of the simulation but do not know the exact time when it is replaced (right censoring). This procedure is done for 1000 replicates for each parameter combination. The generated data is analyzed and compared to the clinical datasets derived from the two prospective cohort studies (Section  2.1).

### 2.4 Estimation of backward mutation rate and relative fitness using a survival function

One of the major challenges in analyzing the backward mutation rate is the difficulty in getting a comprehensive dataset of patients with transmitted drug resistance followed periodically over a long period of time. In most of the observations available, follow-up ends before the replacement of all the resistance-conferring mutations. To make matters worse, the loss to follow-up varies from patient to patient. Thus, information is available about whether or not certain mutations were still present up to a certain time point with no accurate information on when exactly they were replaced.

We seek to define a distribution that can best describe the replacement times of each mutation group. We do this by defining a survival function, S(t), for each mutation group at a given time point. The survival function, S(t), is defined as the probability of a certain resistant mutation surviving at least to time t, that is, the probability that a given resistant mutation is not yet replaced at time t.

We obtain an approximation for the survival function using the Kaplan–Meier estimation (Goel et al., 2010). This estimator makes use of survival times, which measure follow-up time from infection with a resistant virus up until the resistant virus is replaced by a wild type virus. The graph of S(t) against t is called the survival curve. One key advantage of the Kaplan–Meier method is that it can be used to estimate the survival curve from the observed survival times without the assumption of an underlying probability distribution.

A Kaplan–Meier analysis allows for the estimation of the survival over time even when mutations are studied for different lengths of time. At each time point, the survival probability is calculated as follows:

$$S\left(t\right)=\frac{\text{Number of individuals still carrying a given mutation at time }t}{\text{Number of individuals carrying a given mutation at time }t=0}\text{.}$$

The number of mutations that have been replaced, lost to follow up or not reached the time point yet are not counted as being at risk. Mutations that are lost to follow up are considered censored and are not counted in the denominator. The probability of a mutation surviving to any time point is estimated from the cumulative probability of surviving each of the preceding time interval (calculated as a product of preceding probabilities). It is noted that even though the probability at any given interval is not very accurate due to the small number of replacement events, the overall probability of surfing to each point is more accurate.

When interpreting Kaplan–Meier curves, it should be noted that the precision and accuracy of such curves is heavily reliant on the number of observations. In particular, estimates at the left hand side are more precise than at the right hand side because of small numbers due to mutations being replaced or lost to follow up. Kaplan–Meier curves also give often the impression that a mutation replacement occurs more frequently early on than later in time. This is due to the high survival rate and the large number of mutations at the very beginning.

In order to estimate values for the backward mutation rate z and the relative fitness $k_{2}$ for a specific category of mutations, we compare the probability distribution of the replacement times from the data (given by the Kaplan–Meier estimation of the survival function) with that from the stochastic simulations. For given values of z and $k_{2}$ at each time point, the survival function is defined as the proportion, out of the total 1000 replicates, of simulations where the drug-resistant strain is still present.

In order to select the survival function that best fits the data, we define two measures:

(i) m, which represents a (normalized) distance between the survival function for the simulated data and the observed data. It is calculated as
  (2) $$m=\frac{1}{N}\overset{T}{\underset{i=1}{\sum }}{\left(S_{d}\left(t_{i}\right)-S_{s}\left(t_{i}\right)\right)}^{2}$$
  where N is the number of times that replacement events are accounted for during the entire duration of observation, $S_{d}\left(t_{i}\right)$ is the value of the survival function estimated from the clinical data, $S_{s}\left(t_{i}\right)$ is the value of the survival function estimated from the simulation of the stochastic model for a particular pair of values of z and $k_{2}$, and $t_{T}$ is the first time at which no individual in the simulated data carried a particular mutation. For mutations that were not replaced completely amongst the individuals represented by simulated data, we set $t_{T}=3000$.
(ii) n, which represents the percentage of the estimated survival curve from simulated data that is found outside the 99% confidence interval of the observed survival curve, calculated as
  (3) $$n=\frac{\text{No. of time points with }S_{s}\left(t\right)\text{ outside the confidence interval}}{\text{No. of time points with replacement events in clinical data}}100\%\text{.}$$
  It is important to note that it is indeed confidence intervals that are considered, one at each of the times where there are replacement events in the clinical data, rather than confidence bands (for all time points in the window of study). This means that the confidence intervals represent uncertainty in the estimate of the survival function estimated from simulated data at time points with replacement events in the clinical data. Therefore, the validity of these confidence intervals hold only at those time points where replacement events occur in clinical data rather than holding simultaneously for many points.

Both measures, m and n, provide error functions between observed data and simulations. Parameter values that optimize the metrics m and n are found. Given that the metrics are defined in such a way that they measure how close the simulations are to the observed data, such optimized parameter values are good estimates for the optimal parameter values from the data.

## 3 Results

**Fig. 2:**
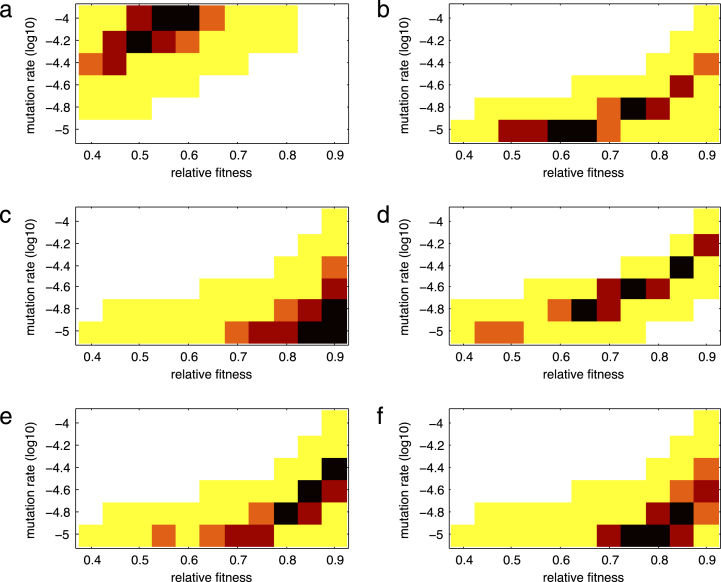
Heat maps for the distance m as a function of the relative fitness k and backward mutation rate z for the drug resistance mutation groups: (a) M184V, (b) TAM, (c) T215 partial revertants, (d) NRTI, (e) NNRTI, and (f) PI. The colors of boxes correspond to the smallest 5%, 10%, 15%, and 50% values of the distance m, for each (k,z) combination, in a red scale (the darker the box, the smaller the value of m). (For interpretation of the references to color in this figure legend, the reader is referred to the web version of this article.)

Fig. 2 shows heat maps for the distance m, defined in (2), between the survival curves estimated from clinical and simulated data for several combinations of values of the relative fitness (k) of a resistant strain and the backward mutation rate (z) for each of the mutation groups. The darker (in a red scale) the box is, the smaller the value of m (colors show the smallest 5, 10, 15, and 50% values of m). The smaller the value of m, the better the estimates of the backward mutation rate and the relative fitness.

**Fig. 3:**
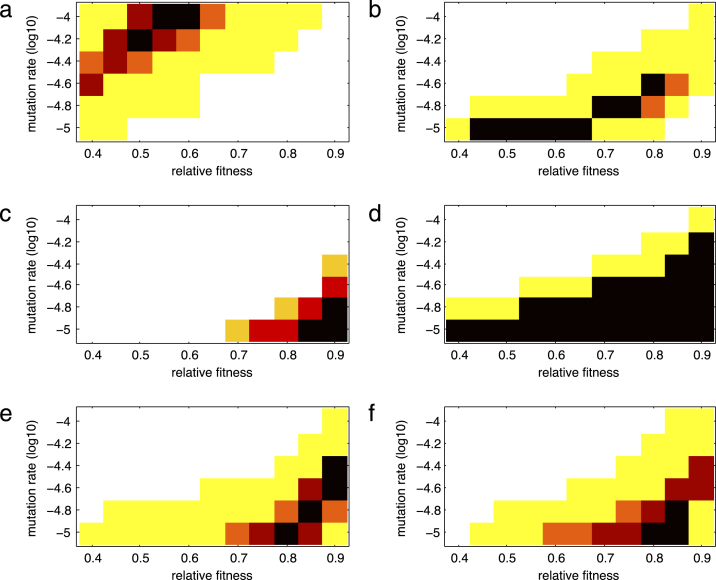
Heat maps for the percentage n of the estimated survival curve outside the 99% confidence interval as a function of the relative fitness k and backward mutation rate z for the drug resistance mutation groups: (a) M184V, (b) TAM, (c) T215 partial revertants, (d) NRTI, (e) NNRTI, and (f) PI. The colors of boxes correspond to the smallest 5%, 10%, 15%, and 50% values of n, for each (k,z) combination, in a red scale (the darker the box, the smaller the value of n). (For interpretation of the references to color in this figure legend, the reader is referred to the web version of this article.)

On the other hand, Fig. 3 shows heat maps for the percentage n of the survival curve estimated from simulated data (defined in (3)) that is found outside the confidence interval of the survival curve estimated from clinical data for different values of the backward mutation rate, z, and the relative fitness, k, of the resistant strain. The darker (in a red scale) the box is, the smaller the value of n (colors show the smallest 5%, 10%, 15%, and 50% values of n). A smaller value of n represents better estimates of the backward mutation rate and the relative fitness.

**Fig. 4:**
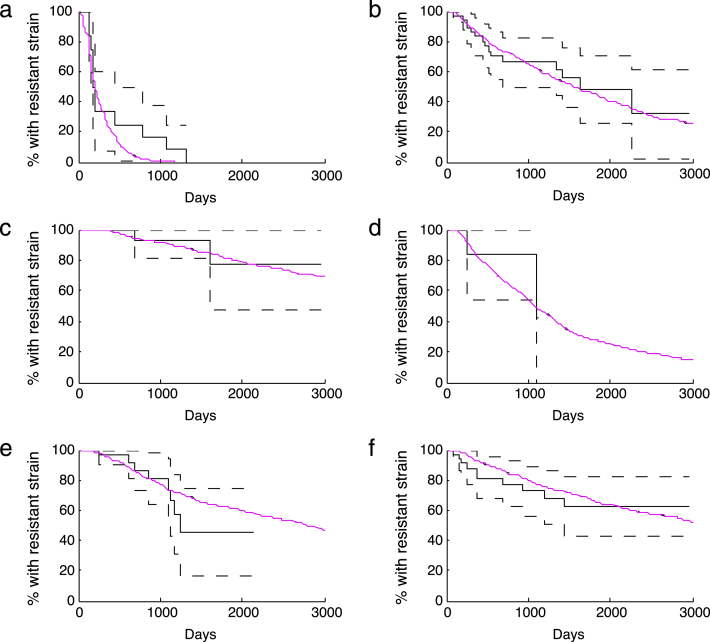
Kaplan–Meier estimation of the survival function over time of the data (solid black; confidence bands as broken curves) and one of the best fitting simulations (purple) for the drug resistance mutation groups: (a) M184V $\left(z=1\times 10^{-4},k=0.55\right)$, (b) TAM $\left(z=1\times 10^{-5},k=0.65\right)$, (c) T215 partial revertants $\left(z=1\times 10^{-5},k=0.90\right)$, (d) NRTI $\left(z=1\times 10^{-4.6},k=0.75\right)$, (e) NNRTI $\left(z=1\times 10^{-4.8},k=0.85\right)$, and (f) PI $\left(z=1\times 10^{-5},k=0.80\right)$. Simulations are drawn for 3000 days, even though in some cases available data correspond to shorter period.

For the Lamivudine/Emtricitabine-associated mutations, the best fitting pairs of values of the backward mutation rate and the relative fitness are found to be (1×10^−4^,0.60),(1×10^−4^,0.55) and (1×10^−4.2^,0.50) as they correspond to 5-percentiles of measures m or n (darker boxes in Figs. 2(a) and/or 3(a)). These results are shown in Table 3. Therefore, a backward mutation rate of 1×10^−4^ and a relative fitness between 0.55 and 0.60 is a good estimation. Alternatively, a lower backward mutation rate of 1×10^−4.2^ would require a reduced relative fitness of 0.5 to achieve a comparable survival curve. This means that for lower values of the backward mutation, lower relative fitness values would be required. Fig. 4(a) shows the Kaplan–Meier estimation of the survival function over time from clinical data and from one of the best fitting simulations ($z=1\times 10^{-4}$ and relative fitness of 0.55) for the Lamivudine/Emtricitabine-associated mutations. The best fitting values of the backward mutation and relative fitness capture the data very well given the fact that a 99% confidence interval is considered for the simulations and the entire simulated curve is found within the confidence band. The general trend of the survival curve of the Lamivudine/Emtricitabine-associated mutations is also well captured, with a 100% probability of Lamivudine/Emtricitabine-associated mutations being replaced during the time of observation (Fig. 4(a)).

For the TAM group, there are several parameter combinations that provide a good fit to clinical data (Figs. 2(b) and 3(b)). These values are summarized in Table 3. In particular, we observe a trade-off between the parameters where a faster rate of backward mutation would require a higher value of relative fitness for a good fit of the data (Figs. 2(b) and 3(b)). Fig. 4b shows the Kaplan–Meier estimation of the survival function over time from clinical data and survival curve resulting from one of the best fitting simulations ($z=1\times 10^{-5}$ and relative fitness of 0.65) for the TAM mutations. The best fitting values of the backward mutation and relative fitness capture the data well given that the entire simulated curve is found within the confidence interval. The general trend of the survival curve estimated from clinical data for the TAM mutations is also well captured (Fig. 4(b)). It is predicted that not all TAM mutations will have been replaced by $t_{T}=3000$ days.

For the T215 partial revertant mutations, a value of 1×10^−5^ for the backward mutation rate and relative fitness lying between 0.85 and 0.9 are found to give a good fit of the clinical data (Figs. 2(c) and 3(c)). Similarly, a backward mutation rate of 1×10^−4.8^ and a relative fitness of 0.9 fits well the observed clinical data. Fig. 4(c) shows the Kaplan–Meier estimation of the observed survival function over time and the survival curve derived from the best fitting simulations ($z=1\times 10^{-5}$ and relative fitness of 0.90) for the T215 partial revertant mutations. Although only around 65% of the simulated curve is found within the confidence interval (because of the size of the available dataset), the general trend of the survival curve of the T215 partial revertant mutations is well captured (Fig. 4(c)). More than 60% of the T215 partial revertant mutations are predicted not to have been replaced by day 3000. This could be mainly the result of having only three replacement events during the entire period of observation for this particular mutation group. Having few replacement events means that the rest of the data is right censored.

For the NRTI mutation group, there is a large combination of parameters that provide a good fit to the observed survival curve (Figs. 2(d) and 3(d)). These values are shown in Table 3. Fig. 4d shows the Kaplan–Meier estimation of the observed survival function over time and one of the best fitting simulations ($z=1\times 10^{-4.6}$ and relative fitness of 0.75) for the NRTI mutations. The simulated survival curve fits the observed survival curve well given that a big portion of the simulated curve lies within the confidence interval (Fig. 4(d)). By day 3000, it is predicted that at least 20% of the NRTI mutations will still be present. However, there is a wide range of possible values for the backward mutation rate and the relative fitness due to the fact that only two replacement events take place during the entire period of observation. Therefore, in estimation of the observed survival curve, the rest of the mutation events are right-censored.

For the NNRTI mutation group, there is also several parameter combinations that provide a good fit of the clinical data (Figs. 2(e) and 3(e)). Table 3 shows these values. A trade-off between the backward mutation rate and the relative fitness is found in this mutation group. Fig. 4(e) shows the Kaplan–Meier estimation of the observed survival function and one of the best fitting simulations ($z=1\times 10^{-4.8}$ and relative fitness of 0.85) for the NNRTI mutations. The best fitting values of the backward mutation and relative fitness capture the data fairly well given that over 98% of the simulated curve is found within the confidence interval. The general trend of the survival function of the NNRTI mutations is also well captured (Fig. 4(e)). We cannot say much about the replacement times since the survival curve does not reach zero, with over 40% of the NNRTI mutations predicted to still be present after 3000 days.

Similarly for the PI mutation class, there are several combinations of parameter values that fit the observed data well (Figs. 2(f) and 3(f)). These estimates are shown in Table 3. Once again, a trade-off between the parameters is observed. Fig. 4(f) shows the Kaplan–Meier estimation of the survival function over time from the clinical data and from one of the best fitting simulations ($z=1\times 10^{-5}$ and relative fitness of 0.80) for the PI mutations. The best fitting values of the backward mutation and relative fitness capture the data well given that the entire simulated curve (at points of replacement) is found within the confidence band. The general trend of the replacement of the PI mutations is also well captured (Fig. 4(f)). It is worth noting that approximately 30% of the PI mutations are predicted to still be present after 3000 days.

### 3.1 Sensitivity analysis

**Fig. 5:**
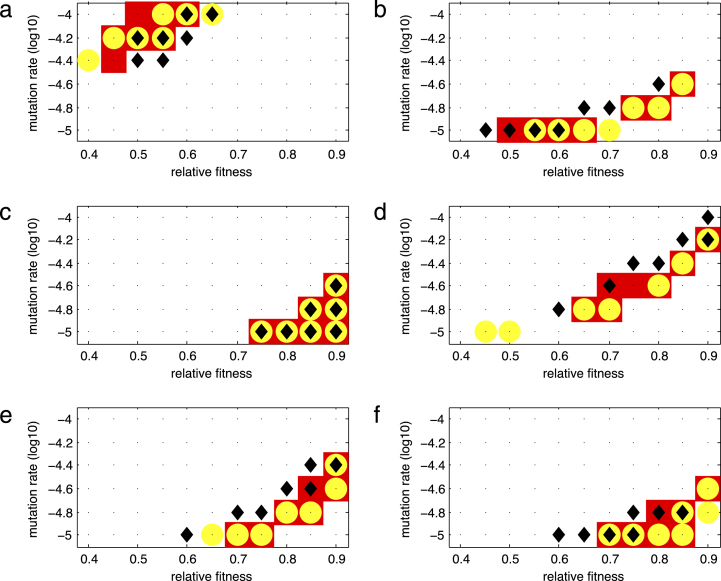
Sensitivity of the infection rate parameter β on the distance m for each of the drug resistance mutation groups: (a) M184V, (b) TAM, (c) T215 partial revertants, (d) NRTI, (e) NNRTI, and (f) PI. The markers in the graphs correspond to smallest 10% values of the distance m for a 50% decrease (black diamond), no change (red box), and a 50% increase (yellow circle) of the baseline value of the parameter β.

The choice of baseline values (Table 2) for some of the parameters were studied. A sensitivity analysis was performed for the infection rate parameter β, where the model’s simulations were run after changing the value of β by either a 50% decrease or a 50% increase of its baseline value. Results for measure m are shown in Fig. 5 (a similar graph was obtained for measure n; see supplementary figure 1). A small shift of the parameter estimates is observed when reducing β by 50% for some of the mutation groups, but not when increasing the value of β. Similarly, for virus production parameter a there is only a small shift of optimal parameter estimates when the value of a is decreased to 50% of its baseline value (supplementary figures 2 and 3).

**Fig. 6:**
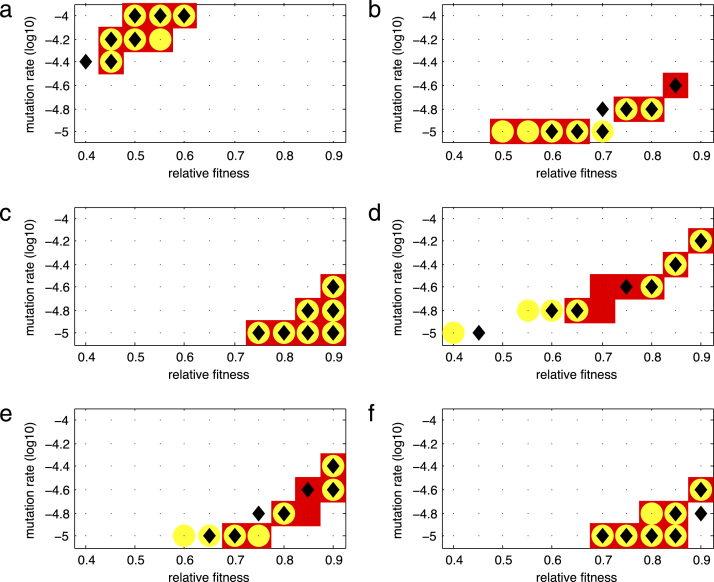
Sensitivity of the initial viral load $V_{r}\left(0\right)$ on the distance m for each of the drug resistance mutation groups: (a) M184V, (b) TAM, (c) T215 partial revertants, (d) NRTI, (e) NNRTI, and (f) PI. The markers in the graphs correspond to smallest 10% values of the distance m for a 50% decrease (black diamond), no change (red box), and a 50% increase (yellow circle) of the baseline value of the parameter $V_{r}\left(0\right)$.

Our choice of initial conditions is similar to values used in previous literature (Cadosh et al., 2012). To study the effect of one of these initial conditions we performed a sensitivity analysis on the inoculum size $V_{r}\left(0\right)$. Fig. 6 shows the best estimates, corresponding to measure m, when the baseline value for $V_{r}\left(0\right)$ is decreased 50% or increased 50% (similar results are obtained for n; supplementary figure 4). In general, there is no significant change on the best estimates for the backward mutation rate and the relative fitness when $V_{r}\left(0\right)$ is varied.

## 4 Discussion and conclusions

In cases of TDR, the replacement of the drug-resistant HIV strain by drug-sensitive HIV as the dominant strain, over time, has been attributed to viral evolution rather than emergence of a pre-existing wild-type variant (Jain et al., 2011). Several transmitted drug resistance-conferring mutations have been reported to persist over a long period of time while others have been reported to be replaced easily by wild-type strains (Barbour et al., 2004, Little et al., 2008). The rate at which a given strain is replaced could depend on a number of factors, both at patient level and at viral level. Viral evolution therefore cannot be assumed to be driven exclusively by selective events (relative fitness and backward mutation) but also by some stochastic forces. By accounting for the stochastic forces, the rate of backward mutation and the relative fitness for each drug mutation class could be good predictors of how quickly, the resistant mutations are replaced. Comparing survival curves derived from clinical data for patients with TDR with survival curves for data generated from a stochastic model of HIV viral dynamics, we have been able to estimate the backward mutation rate and relative fitness of six HIV mutation classes (Table 3). Although for some of the mutation classes, the estimates have considerable uncertainty due to the small datasets.

For the stochastic model, the population size of target cells was taken as 10^4^ cells, which corresponds to modeling the viral dynamics in a volume of 1 ml. This is in agreement to previous modeling work (Cadosh et al., 2012, Pearson et al., 2011, Ribeiro and Bonhoeffer, 2000). However, the effective population size for stochastic within-host models of HIV is still debated (Kouyos et al., 2006). Nevertheless, our definition of replacement (extinction) of the DR strain is constrained by the clinical test sensitivity, which is 50 cells/ml (that is, scaled by ml). Therefore, we do not expect a big difference on the distribution of replacement times as a scaled up of the volume would require a scaled up of the number of cells needed for extinction. Other modeling frameworks different to our approach, *e.g.*  (Heffernan and Wahl, 2005), that consider the stochastic nature of the emergence and persistence of a DS strain and the eventual replacement of the DR strain might also be applicable to study our question.

For the decision of the best fitting survival function to the observed data, we used the distance measure, m, and the percentage of the estimated survival curve from simulated data found outside the 99% confidence interval of the observed survival curve at points of replacement. In general, similar values of the backward mutation rate and relative fitness were predicted by both measures. However, generally the percentage measure, n, had more uncertainty in the estimates. Moreover, we employed the Kaplan–Meier estimation of the survival curve which has the advantage that no probability distribution is assumed for the replacement times of the resistance-conferring mutations. This allowed us to estimate parameters without any unrealistic assumptions on the probability distribution of the replacement times which is still largely unknown. We expect very similar results with alternative measures like the Kullback–Leibler divergence. However, other possibilities, like the log-rank (Mantel–Cox) test, were not ideal as for most of the mutational classes being studied, the replacement events in the clinical data are very few.

Most of the mutation classes had more than one best-fit pair of values for backward mutation rate and the relative fitness, that is, there is uncertainty in the estimates. In particular, we found a trade-off relationship between the backward mutation rate and the relative fitness, where a higher rate of backward mutation requires a higher value of relative fitness to explain the observed data (Fig. 2, Fig. 3). A faster backward mutation could lead to a faster emergence of the wild-type strain. Therefore, a more fit resistant virus is in better position to compete and slow down the emergence of this new strain.

Compared to the other classes, it has been established that the Lamivudine/Emtricitabine-associated mutations have a distinctly higher backward mutation rate and low relative fitness. The higher backward mutation rate and therefore faster replacement is consistent with earlier findings (Barbour et al., 2004, Jain et al., 2011, Little et al., 2008). Similarly for the predicted low relative fitness, as this particular mutation class has been associated with reduced fitness in the absence of treatment (Jain et al., 2011, Paredes et al., 2009).

For the PI mutation group, a high relative fitness (of about 0.08–0.85) was found. This is somewhat surprising due to the fact that, like Lamivudine/Emtricitabine-associated mutations, the PI mutations are associated with reduced viral fitness (Croteau et al., 1997, Martinez-Picado et al., 1999). However, such a finding is consistent with other published reports (Jain et al., 2011, Little et al., 2008). Van Maarseveen et al. (2005) propose compensatory fixation as a mechanism to explain the observed low rate of mutation replacement. Viruses with multiple PI-resistance-conferring mutations partially compensate for the initial loss of fitness.

The fact that PI mutations are replaced at almost similar rates to NNRTI mutations is of clinical significance. It is often argued that in a resource-poor setting, where treatment is initiated without prior resistance testing, a protease inhibitor should be part of the cocktail in the first line treatment options. This is because it is assumed that with its reduced fitness, mutations arising from it can be easily replaced by wild-type strains presenting a lower risk of TDR propagation. However, having a high fitness, as our findings suggest, may in fact increase the risk of TDR. This would be the result of the resistant strain persisting long enough in treatment-naïve patients, increasing the chances of being spread through populations. Resistance propagation is worsened in resource-limited settings where ART is initiated without drug resistance testing. In such settings, patients could be initiated on a treatment regimen that is partially effective and exposed to it over a prolonged period, which would lead to the spread of the resistant strain and the possibility of additional drug resistance-conferring mutations being selected, resulting into having limited options for second-line treatment.

Having knowledge of the backward mutation rate for different mutation groups could help clinicians interpret resistance tests performed during the chronic stage. While interpreting the resistance test results, it could be important to note that some mutation classes such as the Lamivudine/Emtricitabine-associated mutations could have been greatly reduced due to the faster replacement with the wild type at the time of testing (Jain et al., 2011). They could still be present at minority levels that are undetectable by standard tests. Even for mutation classes such as NNRTI and PI with high relative fitness predicted, it should be noted that these can also be replaced over time given the background rate of backward mutations that can lead to emergence of a slightly fitter wild-type strain. Once a fitter strain has emerged, it is then a question of competition between the two strains that determines the dominant strain (Kitayimbwa et al., 2013, Rong et al., 2007, Vaidya et al., 2010).

## Figures and Tables

**Table 1:** Events and corresponding probabilities for the stochastic model.

Event	Change in population size t→t+Δt	Probability
Recruitment of T	T→T+1	λΔt/Φ
Death of T	T→T−1	γTΔt/Φ
Infection by $V_{s}$	$T\to T-1,\;I_{s}\to I_{s}+1$	$\beta TV_{s}\Delta t/\Phi$
Infection by $V_{r}$	$T\to T-1,\;I_{r}\to I_{r}+1$	$\left(1-z\right)k_{1}\beta TV_{r}\Delta t/\Phi$
Infection by $V_{r}$ mutating to $V_{s}$	$T\to T-1,\;\;I_{s}\to I_{s}+1$	$zk_{1}\beta TV_{r}\Delta t/\Phi$
Death of $I_{s}$	$I_{s}\to I_{s}-1$	$\delta I_{s}\Delta t/\Phi$
Death of $I_{r}$	$I_{r}\to I_{r}-1$	$\delta I_{r}\Delta t/\Phi$
Production of $V_{s}$	$V_{s}\to V_{s}+1$	$aI_{s}\Delta t/\Phi$
Production of $V_{r}$	$V_{r}\to V_{r}+1$	$k_{2}aI_{r}\Delta t/\Phi$
Death of $V_{s}$	$V_{s}\to V_{s}-1$	$cV_{s}\Delta t/\Phi$
Death of $V_{r}$	$V_{r}\to V_{r}-1$	$cV_{r}\Delta t/\Phi$

**Table 2:** Parameter values used in simulations.

Parameter	Definition	Value	Reference
$T_{0}$	Initial target cell count	1.5×10 ^4^ cells/ml	Buckley and Gluckman (2002)
γ	Death rate of target cells	0.01 d^−1^	Mohri et al. (1998)
λ	Recruitment rate of target cells	1.5×10 ^2^ cells/ml d^−1^	Defined as $\gamma T_{0}$
β	Infection rate of target cells by $V_{s}$	6.5×10^−7^ ml d^−1^	Perelson et al. (1993)
$k_{1}$	Relative fitness of $V_{r}$ infectivity	1	–
δ	Death rate of infected cells	0.39 d^−1^	Vaidya et al. (2010)
a	Rate of virus production	850 (cells/ml)${}^{-1}$d^−1^	Vaidya et al. (2010)
$k_{2}$	Relative fitness of $V_{r}$ replication	varied	–
c	Clearance rate of free virus	3 d^−1^	Ramratnam et al. (1999)
z	Backward mutation rate	varied	–

**Table 3:** Estimated backward mutation rates and relative fitness for the different mutation classes. Estimates shown correspond to smallest 5% values of either measure m or n.

Mutation class	Backward mutation rate	Relative fitness
Lamivudine/emtricitabine-	1×10^−4^	[0.55,0.60]
associated mutations	1×10^−4.2^	0.50
TAM	1×10^−5^	[0.45,0.65]
	1×10^−4.8^	[0.70,0.75]
	1×10^−4.6^	0.80
T215 partial revertant	1×10^−5^	[0.85,0.90]
mutations	1×10^−4.8^	0.90
NRTI mutations	1×10^−5^	[0.40,0.90]
	1×10^−4.8^	[0.55,0.90]
	1×10^−4.6^	[0.70,0.90]
	1×10^−4.4^	[0.85,0.90]
	1×10^−4.2^	0.90
NNRTI mutations	1×10^−5^	0.80
	1×10^−4.8^	[0.80,0.85]
	1×10^−4.6^	[0.85,0.90]
	1×10^−4.4^	0.90
PI mutations	1×10^−5^	[0.75,0.85]
	1×10^−4.8^	0.85

## Appendix A Supplementary data

MMC S1 Sensitivity of the infection rate parameter β on the distance n for each of the drug resistance mutation groups: (a) M184V, (b) TAM, (c) T215 partial revertants, (d) NRTI, (e) NNRTI, and (f) PI. The markers in the graphs correspond to smallest 10% values of the distance n for a 50% decrease (black diamond), no change (red box), and a 50% increase (yellow circle) of the baseline value of the parameter β.

MMC S2 Sensitivity of the infection rate parameter a on the distance m for each of the drug resistance mutation groups: (a) M184V, (b) TAM, (c) T215 partial revertants, (d) NRTI, (e) NNRTI, and (f) PI. The markers in the graphs correspond to smallest 10% values of the distance m for a 50% decrease (black diamond), no change (red box), and a 50% increase (yellow circle) of the baseline value of the parameter a.

MMC S3 Sensitivity of the infection rate parameter a on the distance n for each of the drug resistance mutation groups: (a) M184V, (b) TAM, (c) T215 partial revertants, (d) NRTI, (e) NNRTI, and (f) PI. The markers in the graphs correspond to smallest 10% values of the distance n for a 50% decrease (black diamond), no change (red box), and a 50% increase (yellow circle) of the baseline value of the parameter a.

MMC S4 Sensitivity of the infection rate parameter $V_{r}\left(0\right)$ on the distance n for each of the drug resistance mutation groups: (a) M184V, (b) TAM, (c) T215 partial revertants, (d) NRTI, (e) NNRTI, and (f) PI. The markers in the graphs correspond to smallest 10% values of the distance n for a 50% decrease (black diamond), no change (red box), and a 50% increase (yellow circle) of the baseline value of the parameter $V_{r}\left(0\right)$.
