# Supplementary figures and images for: Estimation of the HIV-1 backward mutation rate from transmitted drug-resistant strains
Source: Theor Popul Biol. 2016 Dec;112:33–42. doi: 10.1016/j.tpb.2016.08.001 (PMC5126109; doi:10.1016/j.tpb.2016.08.001)

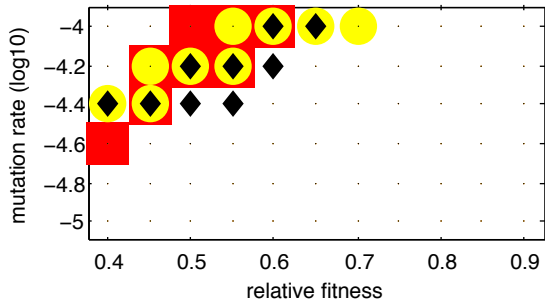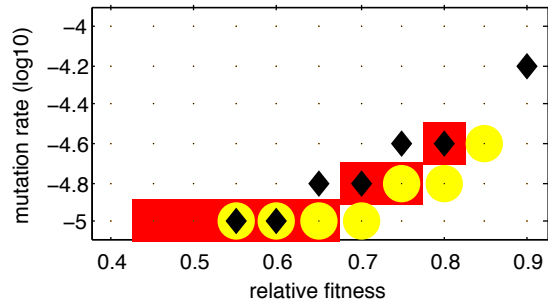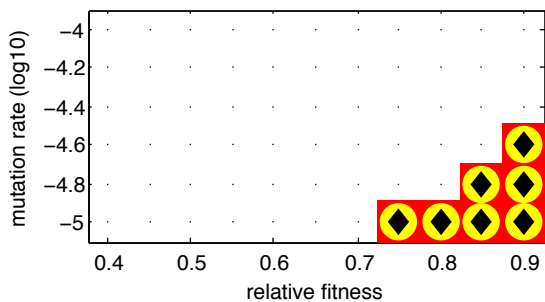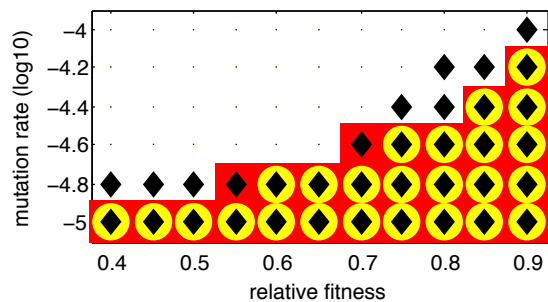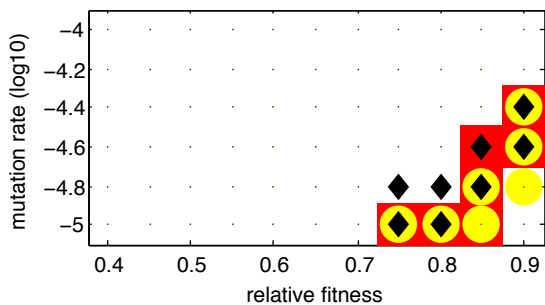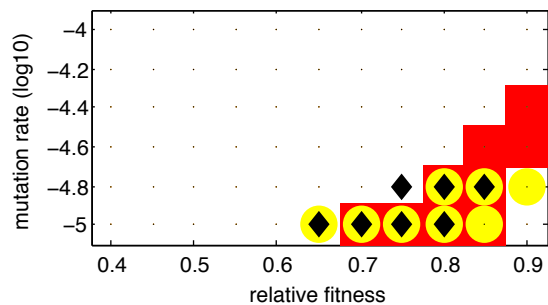

Supplement: MMC S1 — Sensitivity of the infection rate parameter β on the distance n for each of the drug resistance mutation groups: (a) M184V, (b) TAM, (c) T215 partial revertants, (d) NRTI, (e) NNRTI, and (f) PI. The markers in the graphs correspond to smallest 10% values of the distance n for a 50% decrease (black diamond), no change (red box), and a 50% increase (yellow circle) of the baseline value of the parameter β. [file mmc1.pdf]

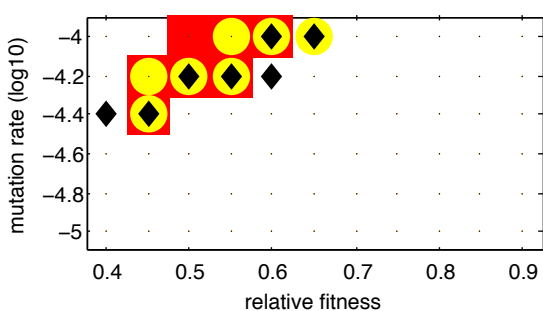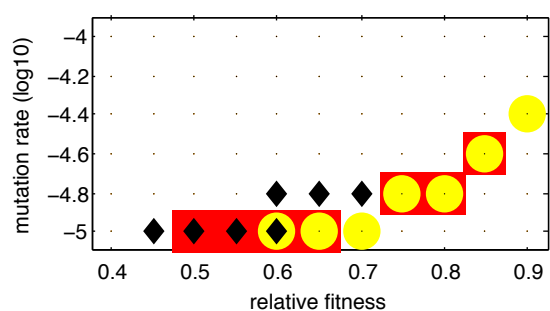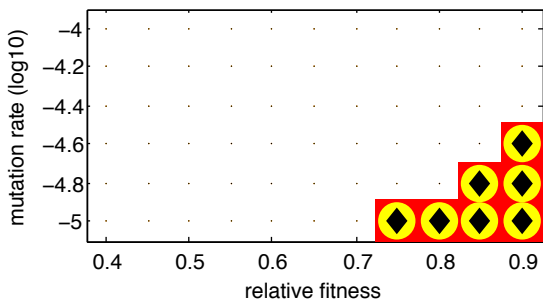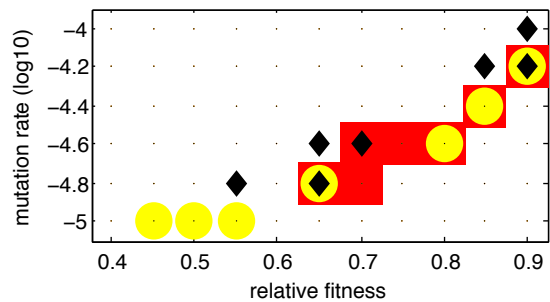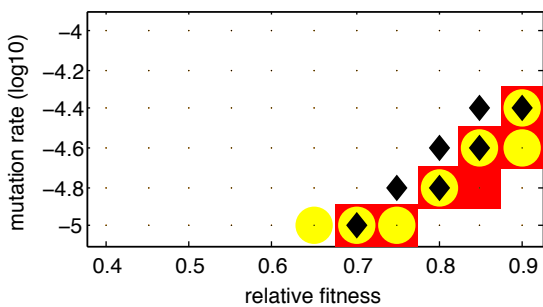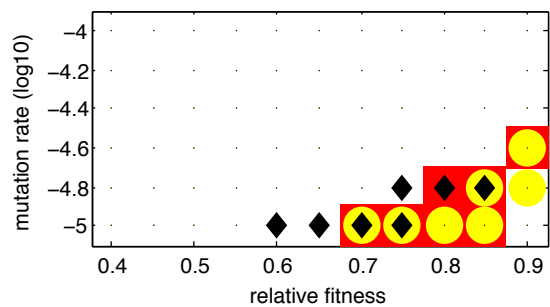

Supplement: MMC S2 — Sensitivity of the infection rate parameter a on the distance m for each of the drug resistance mutation groups: (a) M184V, (b) TAM, (c) T215 partial revertants, (d) NRTI, (e) NNRTI, and (f) PI. The markers in the graphs correspond to smallest 10% values of the distance m for a 50% decrease (black diamond), no change (red box), and a 50% increase (yellow circle) of the baseline value of the parameter a. [file mmc2.pdf]

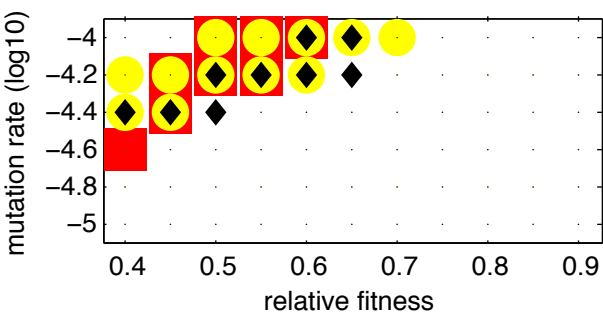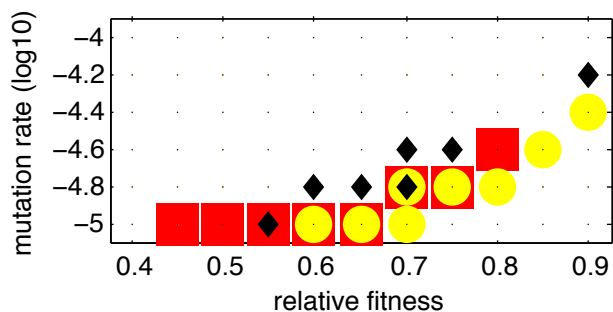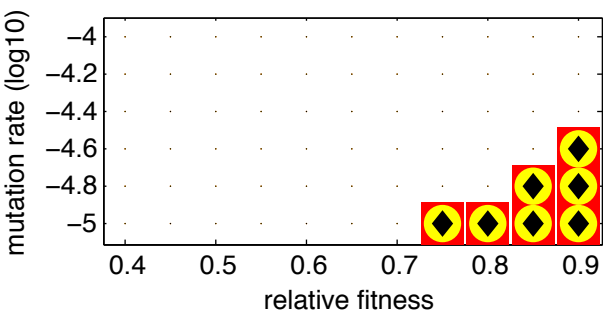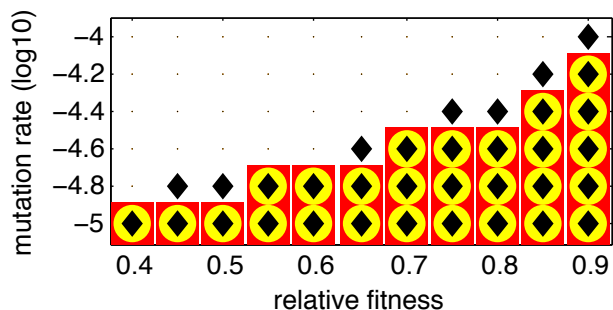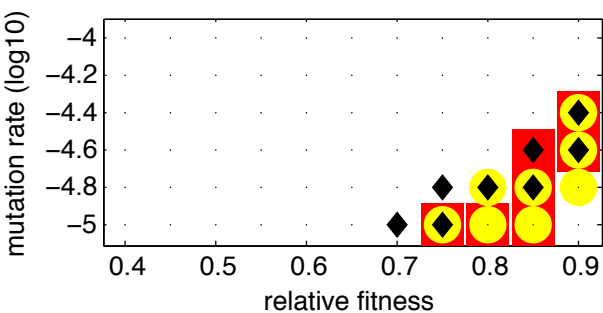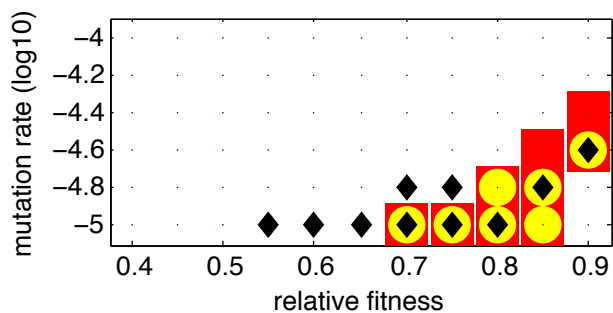

Supplement: MMC S3 — Sensitivity of the infection rate parameter a on the distance n for each of the drug resistance mutation groups: (a) M184V, (b) TAM, (c) T215 partial revertants, (d) NRTI, (e) NNRTI, and (f) PI. The markers in the graphs correspond to smallest 10% values of the distance n for a 50% decrease (black diamond), no change (red box), and a 50% increase (yellow circle) of the baseline value of the parameter a. [file mmc3.pdf]

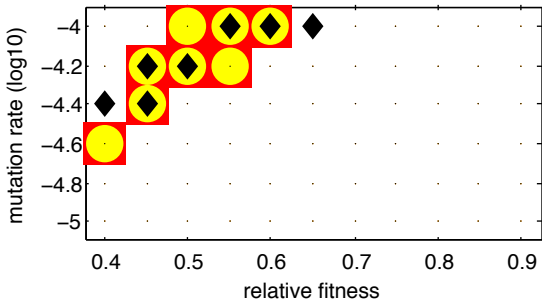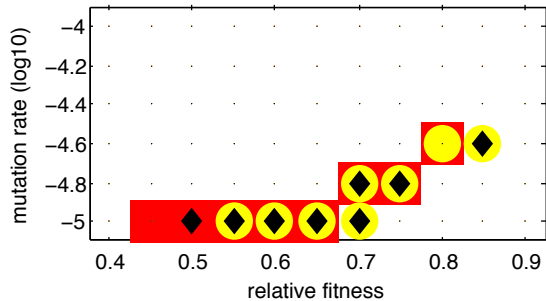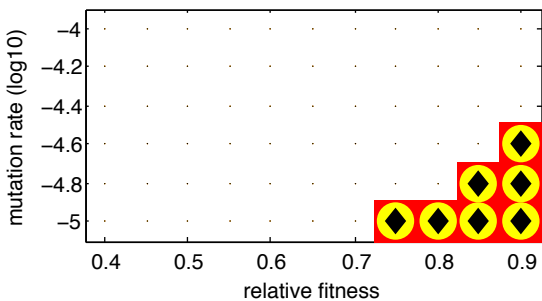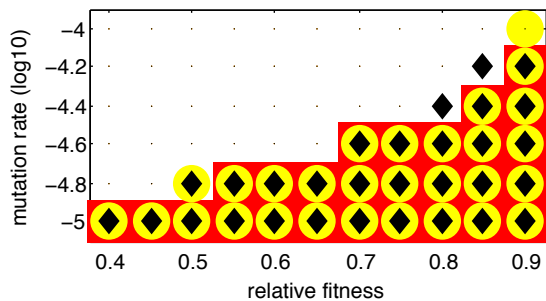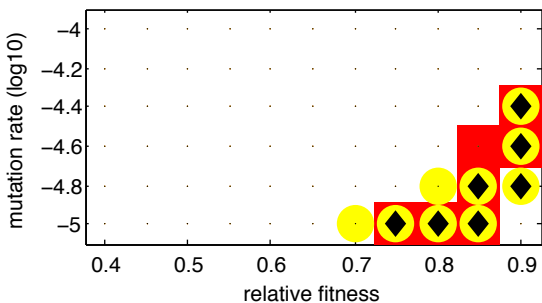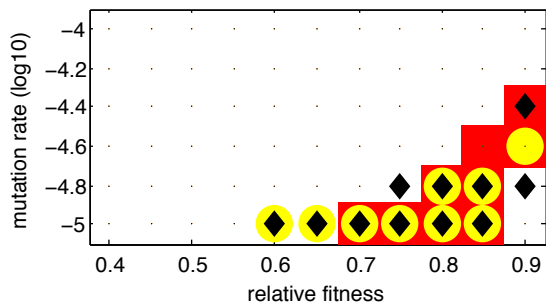

Supplement: MMC S4 — Sensitivity of the infection rate parameter Vr(0) on the distance n for each of the drug resistance mutation groups: (a) M184V, (b) TAM, (c) T215 partial revertants, (d) NRTI, (e) NNRTI, and (f) PI. The markers in the graphs correspond to smallest 10% values of the distance n for a 50% decrease (black diamond), no change (red box), and a 50% increase (yellow circle) of the baseline value of the parameter Vr(0). [file mmc4.pdf]
